# Expansion of the Tibetan Plateau during the Neogene

**DOI:** 10.1038/ncomms15887

**Published:** 2017-06-21

**Authors:** Weitao Wang, Wenjun Zheng, Peizhen Zhang, Qiang Li, Eric Kirby, Daoyang Yuan, Dewen Zheng, Caicai Liu, Zhicai Wang, Huiping Zhang, Jianzhang Pang

**Affiliations:** 1 State Key Laboratory of Earthquake Dynamics, Institute of Geology, China Earthquake Administration, Beijing 100029, China; 2 School of Earth Science and Geological Engineering, Sun Yan-Sen University, Guangzhou 510275, China; 3 Institute of Vertebrate Paleontology and Paleoanthropology, Chinese Academy of Sciences, Beijing 100044, China; 4 College of Earth, Ocean and Atmospheric Sciences, Oregon State University, Corvallis, Oregon 97330, USA; 5 Lanzhou Institute of Seismology, China Earthquake Administration, Lanzhou 730000, China; 6 Institute of Earthquake Engineering, Shandong Earthquake Administration, Jinan 250014, China

## Abstract

The appearance of detritus shed from mountain ranges along the northern margin of the Tibetan Plateau heralds the Cenozoic development of high topography. Current estimates of the age of the basal conglomerate in the Qaidam basin place this event in Paleocene-Eocene. Here we present new magnetostratigraphy and mammalian biostratigraphy that refine the onset of basin fill to ∼25.5 Myr and reveal that sediment accumulated continuously until ∼4.8 Myr. Sediment provenance implies a sustained source in the East Kunlun Shan throughout this time period. However, the appearance of detritus from the Qilian Shan at ∼12 Myr suggests emergence of topography north of the Qaidam occurred during the late Miocene. Our results imply that deformation and mountain building significantly post-date Indo-Asian collision and challenge the suggestion that the extent of the plateau has remained constant through time. Rather, our results require expansion of high topography during the past 25 Myr.

## Abstract (web summary)

The timing of mountain building along the Tibetan Plateau remains unclear. Here, the authors present new magnetostratigraphic and mammalian biostratigraphic data from sediments to show that mountain building at the edge of the Tibetan Plateau commenced at 25.5 Ma with a separate emergence in the north at 12 Ma.

Time-space patterns of deformation within the Eurasian continent reflect the evolution of high topography associated with the Indo-Asian collision^1^,^2^,^3^. Although most models predict that the locus of crustal thickening and deformation should expand with progressive convergence between India and Eurasia^4^,^5^ several recent studies suggest that regions far inboard from the collision experienced deformation early in the collision history^6^,^7^,^8^,^9^,^10^. These two scenarios imply quite different geodynamics during intracontinental deformation. In the former view, systematic expansion of the region of deformation and mountain building reflects a transfer of potential energy through thickened lithosphere from an initial collisional boundary^4^,^5^. In the latter view, simultaneous deformation in northeast Tibet and the onset of India-Asian collision have been suggested to reflect a relatively constant bulk strain rate of Eurasian lithosphere through time^11^.

In northeast Tibet, the onset of mountain building is considered to have initiated in early Eocene time (50–45 Myr), based on three sources of proxy data. First, low-temperature thermochronology from the hanging wall blocks of major thrust systems suggest that cooling during erosion marks the development of topography above active faults^9^,^10^. Second, the accumulation of coarse, clastic detritus shed from rapidly eroded mountain ranges^6^,^8^,^12^ suggests the development of topographic relief. Third, vertical-axis rotation of these sedimentary deposits calculated from the paleomagnetic declination with respect to the APWP of Eurasia^7^,^13^ suggest a clockwise rotation in Paleogene time.

The >10 km thick Cenozoic deposits in the Qaidam basin (Fig. 1a) play an outsized role in this debate^14^,^15^. These Cenozoic strata have been subdivided into seven primary stratigraphic units: the Lulehe, Xia Ganchaigou, Shang Ganchaigou, Xia Youshashan, Shang Youshashan, Shizigou and Qigequan formations^6^,^12^,^14^,^15^. The basal Cenozoic strata (termed the Lulehe Fm.) consist of a coarse wedge of conglomerate whose provenance reflects the bedrock ranges adjacent to the basin. Most workers agree that the character of the sediment, its provenance, and its geometry relative to the basin margins suggest that it was derived in response to the onset of mountain building in northern Tibet^6^,^12^,^16^. Despite its tectonic significance, the age of Lulehe Fm. is poorly constrained, and, based largely on pollen and ostracode assemblages, is considered to be Paleocene-early Eocene in age^6^,^14^,^17^. This association in time with the Indo-Asian collision^18^,^19^ has led most workers to consider that mountain building around the margins of the Qaidam basin was a far-field response to continental collision^6^,^12^.

In this study, guided by the recognition of a new mammalian fossil assemblage, we reevaluate the age of the Lulehe Fm. in the Honggou section, along the northern margin of the Qaidam basin^20^,^21^,^22^ (Fig. 1a). Using high resolution magnetostratigraphy, we refine and revise the chronology of sediment accumulation in the northern Qaidam basin. We discovered fossil mammals from stratigraphic formations that overlie the Lulehe Fm., which in combination with magnetostratigraphic correlation to the Geomagnetic Polarity Timescale demonstrates that the Lulehe Fm. is Oligocene in age, significantly younger than the previously assumed^6^,^12^,^14^,^20^,^21^,^22^,^23^.

## Results

### Stratigraphy of the Honggou section

**Figure 1:**
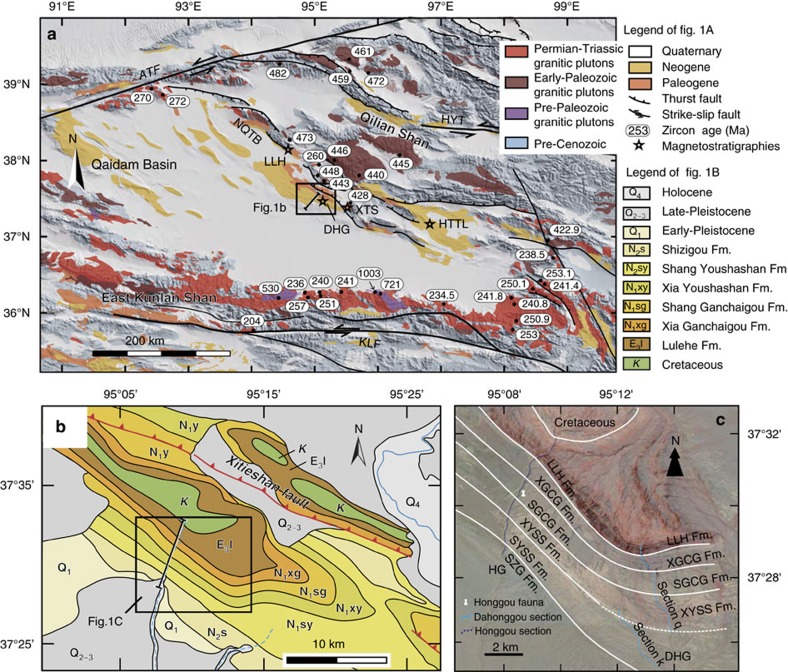
Generalized tectonic and topographic maps of the study region. (**a**) Geological map of the Qaidam basin showing extent of basin fill, major faults, and granitic pluton ages around the Qaidam basin. NQTB:North Qaidam Thrust Belt; ATF: Altyn Tagh Fault; KLF:Kunlun Fault; HYT: Haiyuan Fault; LLH, Lulehe stratigraphic section; DHG, XTS and HTTL, Dahonggou, Xitie Shan and Huaitoutala magnetostratigraphic sections, respectively. (**b**) Geological map of the Honggou anticline showing location of the Honggou magnetostratigraphic section. (**c**) Google Earth image (Google earth V 7.1.2.2041, February First 2005, Eye alt 18 km) of the Honggou region, location of Honggou fauna and stratigraphic correlations in the study region.

The Honggou section is exposed in a fault-related anticline along the northern margin of the basin (Fig. 1). Along the southern limb of the structure in the footwall of the Xitieshan fault, nearly 5.3 km of Cenozoic strata are continuously exposed in a southwest-dipping homocline (Fig. 1b). Previous study of the Dahongou section (including sections q and k) sampled the middle ∼3 km of these strata, including the Shang Ganchaigou, Xia Youshashan, and Shang Youshashan formations (Fig. 1), and suggested that the paleomagentic reversal stratigraphy of the Shang Ganchaigou Fm. could be correlated to GPTS in the Oligocene^20^. We studied a longer and more continuous section father west along the Honggou river valley (extending from 37°32′25.2" N, 95°10′5.57" E to 37°28′52.7" N, 95° 8′5.9" E, Fig. 1b,c). Here sedimentologic characteristics and detrital zircon provenance studies suggest a shift in sedimentary source from the East Kunlun Shan in the lower strata to sources in the Qilian Shan in the upper strata^16^. We describe the stratigraphy with a summary of the most common lithofacies (Supplementary Table 1) below briefly, as a foundation to our paleomagnetic study.

**Figure 2:**
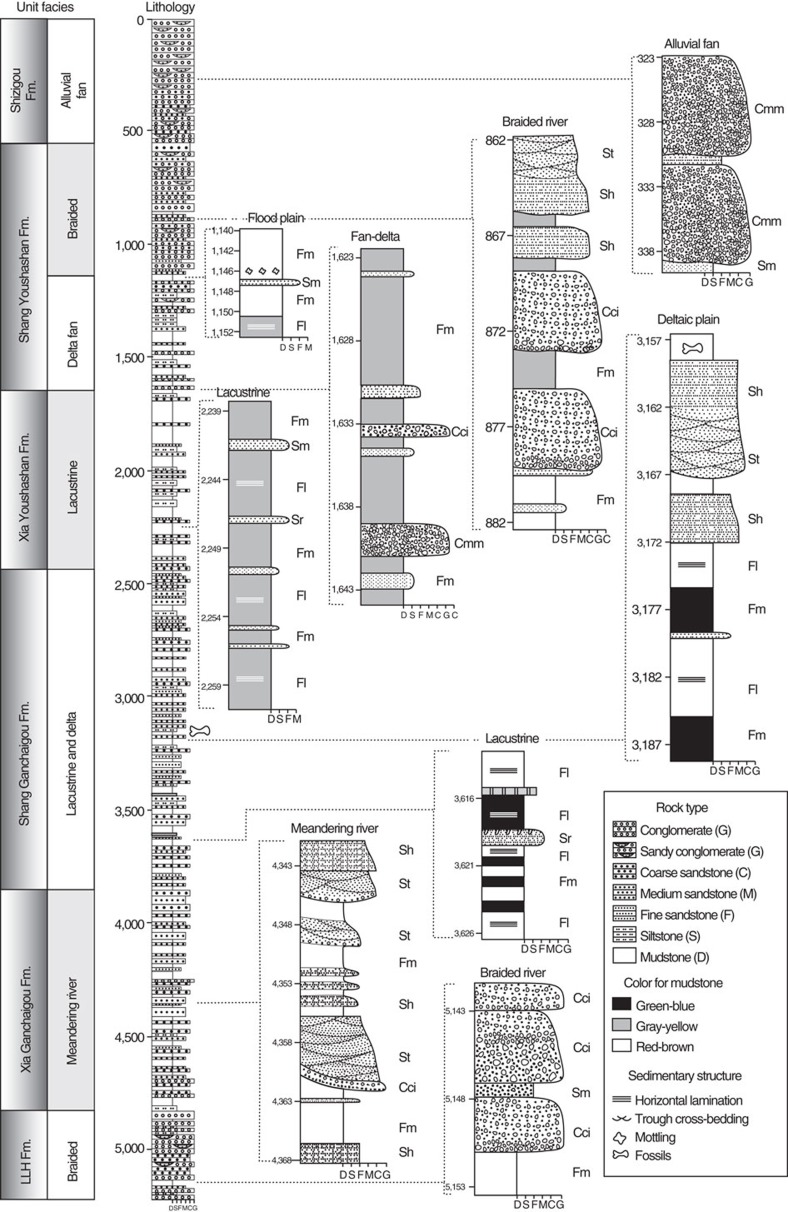
Lithologies and interpreted depositional facies of the Honggou section. The Honggou section was subdivided into Lulehe, Xia Ganchaigou, Shang Ganchaigou, Xia Youshashan, Shang Youshashan and Shizigou Formations. Expanded columns illustrate major facies for each stratigraphic unit within the Honggou section. Facies code abbreviations are described in Supplementary Table 1.

Along the Honggou section, the Lulehe Fm. is 490 m thick and unconformably overlies lower Cretaceous dark-red beds. This formation is predominantly purple to brick red conglomerate intercalated with red sandy mudstone and siltstone (Fig. 2). The conglomerate beds in the Lulehe Fm. are typically massive, clast-supported, and exhibit erosive contacts with underlying units. Clasts within conglomerate beds are 2–20 cm in size, poorly sorted and subrounded to subangular. Massive siltstones and mudstones appear to be tabular at the scale of an outcrop, but they pinch out laterally, over length scales of tens of meters. We interpret the Lulehe Fm. to have accumulated mainly in a braided fluvial system based on the presence of erosional contacts, upward fining sequences and trough-cross bedding within the sandstones^24^,^25^ (Fig. 2).

The uniform purple to brick red colour and poor sorting of conglomerates are the most significant features of the Lulehe Fm., which make it easy to distinguish from underlying and overlying strata. These characteristics are the primary basis for lithostratigraphic correlation to its type Lulehe section^14^, ∼100 km west of the Honggou section and corresponding stratigraphic unit designations (Lulehe Fm.) in the Dahonggou section^21^ and Xitieshan section^22^ (Fig. 1a).

The 980 m thick Xia Ganchaigou Fm. contains green to yellow, sandstones, siltstones and red to green mudstones (Fig. 2). Sandstones within this unit are 5–40 m thick and display lenticular shape with erosional bases. Sedimentary structures include trough cross-stratification and parallel-lamination. The sandstones show upward-fining trend; they are probably produced by the lateral accretion in the point bar as the meander migrates^26^,^27^,^28^. Finer deposits in the Xia Ganchaigou Fm. are 2–5 m thick, purple red to green mudstone, siltstone and very fine-grained sandstone beds (Fig. 2). Small scale cross-stratification and finer horizontal-lamination can be observed in these finer deposits. We interpret the Xia Ganchaigou Fm. to have accumulated in a meander river system. The cross-bedding, erosional contacts of the coarse sandstones and lens-shape geometric conglomerates reveal that many of the beds were deposited by relatively energetic currents^26^. The finer deposits with laminations may be deposited in crevasse splay or fairly still water on floodplain environment^24^,^29^.

The 1,400 m thick Shang Ganchaigou Fm. is composed of variable coloured mudstone, yellowish sandstone and thin limestone (Fig. 2). Sandstones within the Shang Ganchaigou Fm. are typically medium to fine grained, containing trough cross-stratifications, parallel-lamination and rare ripple mark. Mudstones are red to green, 2–15 m thick, tabular, and laterally continuous over hundreds of meters. The marl beds are 0.1–0.4 m thick and occur in tabular shape. These rocks are yellow- to white-weathering, gray micrite to microsparite, with abundant of nodules from 5–10 mm in size. Finer sporadic ripples and horizontal laminations are preserved in these marls. Considering the fact that thin marl beds imply rise of the lake level to highstand^30^,^31^; the thick laminated mudstones suggest mud flat, or lacustrine deposition, and the thick sandstones may represent channel deposits^24^, we interpreted the Shang Ganchaigou Fm. as the deposits of shallow lacustrine and delta (Fig. 2).

The Xia Youshashan Fm. is 820 m thick and is dominated by light brown mudstones and siltstones (Fig. 2). Mudstone beds within the Xia Youshashan Fm. are 5–75 m thick and laterally continuous over hundreds of meters. Relatively thin yellow sandstone and gray conglomerate beds are intercalated within thick mudstones. Horizontal-laminations can be observed in the siltstones and mudstones, whereas trough-cross bedding and parallel bedding are strictly limited in the sandstone beds. The Xia Youshashan Fm. was interpreted as lacustrine deposits due to dominantly thick, laminated mudstones and siltstones^32^,^33^ (Fig. 2).

The Shang Youshashan Fm. is 1070, m thick and is characterized by interbedded mudstone, gravel sandstone and conglomerate (Fig. 2). Clast-supported conglomerates in the lower part of the Shang Youshashan Fm. are found in lenses 0.1–0.3 m thick. These beds are sandwiched within finer facies to make up the bulk of the sequences in place where it is closely associated with horizontal-bedded gravelly sandstone. Conglomerates within the upper part of the Shang Youshashan Fm. are clast-supported, poorly sorted and subrounded to angular. We interpreted the coarser conglomerates as channel products based on imbrication fabrics and erosive bases^24^,^26^. The well-preserved fine lamination, thick fine-textured deposits in the lower part of the Shang Youshashan Fm. are interpreted to deposit in the lacustrine environment^31^. Given its limited distribution and spatial variations of the facies sequences, we infer that the Shang Youshashan Fm. might represent lacustrine and delta in the lower part and braided river in the upper part of the Shang Youshashan Fm. (Fig. 2).

The Shizigou Fm. is 550 m thick and contains pebble, cobble, clast-supported and matrix-supported conglomerates (Fig. 2). Clast-supported conglomerates are 0.5–2 m thick, whereas matrix-supported conglomerates are 2–18 m thick, with lenticular geometry and disorganized texture. Both of the conglomerate lithofacies contain poorly sorted, angular to subrounded clasts. The clast-supported conglomerates exhibit lenticular geometry and erosive bases, suggesting that they were deposited as channel fills^26^, and the matrix-supported conglomerates may have originated from gravity flows^25^. Therefore, the deposits of the Shizigou Fm. may represent an alluvial fan environment.

### New fossil assemblage from the Honggou section

**Figure 3:**
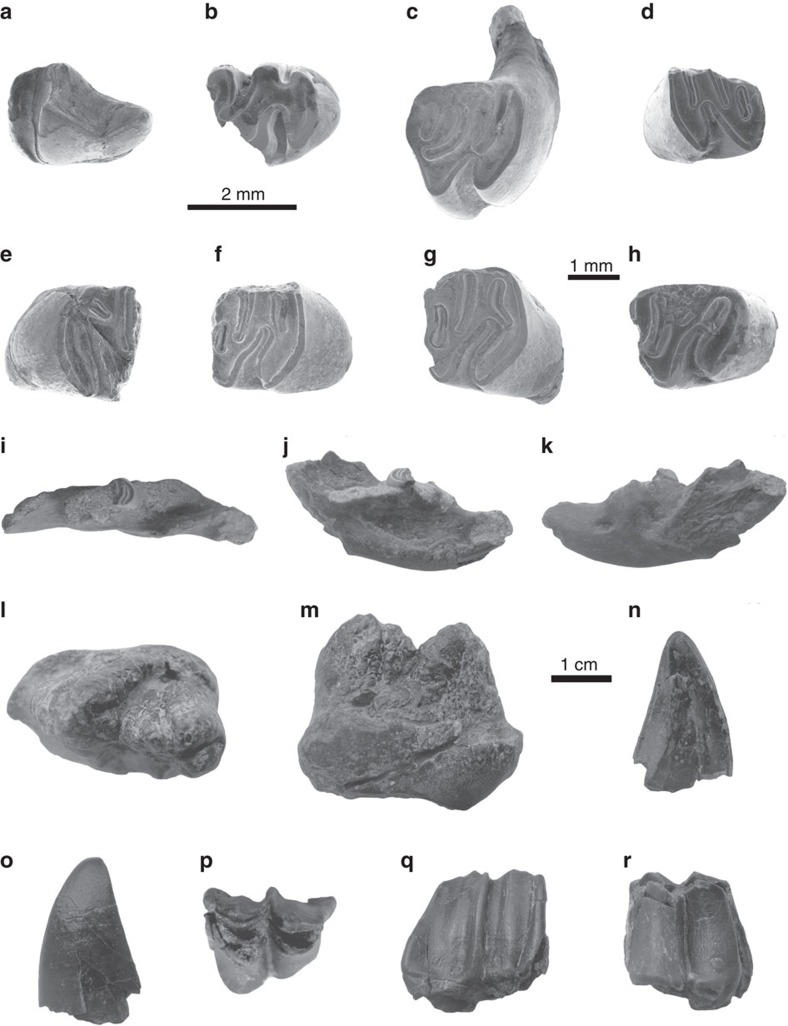
Fossil mammalians of the Honggou fauna. (**a**) *Mioechinus*? sp., right lower fourth premolar (p4). (**b**) *Pleisodipus* sp., left lower third molar (m3). (**c**–**k**) *Monosaulax tungurensis* (**c**: right lower fourth premolar (p4); (**d**,**e**) left lower first or second molar (m1/m2); (**f**,**g**) left lower first or second molar (m1/m2); (**h**: left lower third molar (m3); **i**–**k**: left lower jaw with first molar). (**l**,**m**) *Zygolophodon* sp., anterior part of a cheek tooth. (**n**,**o**) Rhinocerotidae indet (broken left lower canine). (**p**–**r**) *Turcocerus* sp., left upper third molar (M3). In the figure, **a** and **b** are in the same scale (scale bar, 2 mm); **c**–**h** are in the same scale (scale bar, 1 mm); **i**–**r** are in the same scale (scale bar, 1 cm).

Within the middle part of the Shang Ganchaigou Fm. (the level of 3,158 m in the Honggou section), we recovered abundant mammalian fossils which are referred to as the Honggou fauna. Six taxa can be recognized from the Honggou fauna; these are *Mioechinus*? sp., *Monosaulax tungurensis*, *Plesiodipus* sp., *Zygolophodon* sp., *Turcocerus* sp., and Rhinocerotidae indet (Fig. 3). The following descriptions are identifications of the recovered mammal materials and their age affinities.

*Mioechinus*? sp.: A right p4 is present (Fig. 3a). It has an anteriorly elongated paraconid and not very lophid metaconid. In overall shape and size, it is very similar to right p4 of *Mioechinus*? sp. from the Tunggur Fm. in Inner Mongolia^34^.

*Plesiodipus* sp.: Only an anterior part broken left m3 is available, which is sufficient to demonstrate its presence in the fauna (Fig. 3b). This tooth has a relatively low crown, but it is not very narrow. Size and crown morphology are closer to that of *Pleisodipus leei* from the Tunggur Formation^34^.

*Monosaulax tungurensis*: Seven specimens have been collected, including a left jaw with fragmentary m1 and one right p4, two left m1/m2s, one left m3 (Fig. 3c-k). Chinese *Monosaulax* includes only two species and both of them are middle Miocene in age; *M. changpeiensis* from Zhangbei, Hebei Province and *M. tungurensis* from the Tunggur fauna in Inner Mongolia^35^. On the basis of the characteristics of robust lower jaw, relatively high crowned cheek teeth, deep hypostriid and similar size with above mentioned *M. tungurensis*, we assign these fossils to *M. tungurensis.*

*Zygolophodon* sp: An anterior part of a cheek tooth has been collected (Fig. 3l,m). It is small in size with low crown height and bunodont structure, suggesting a primitive *Zygolophodon* gomphothere.

Rhinocerotidae indet: Only a section of a robust lower canine is available (Fig. 3n,o). This lower canine shows the presence of a rhino from the Honggou fauna.

*Turcocerus* sp: We have collected a nearly complete left M3 in the Honggou fauna (Fig. 3p-r). Size and morphology are both consistent with the holotype (AMNH 26508) of *Turcocerus grangeri* from Tunggur Fm. in Inner Mongolia^36^. For lack of additional diagnostic material, we cannot further identify this taxon.

Terrestrial Neogene faunas from China have been subdivided into 16 typical local faunas^37^ (Supplementary Fig. 1). For these 16 faunas, Tunggur fauna is a well know middle Miocene mammal assemblage with fossils of more than 30 species^37^. All six taxa of the Honggou fauna discovered in the 3,158 m along the Honggou section are consistent with those from the middle Miocene Tunggur assemblage in Inner Mongolia^37^,^38^, but have little in common with the early-middle Miocene Lengshuigou fauna and late Miocene Tuosu faunas^37^ (Supplementary Fig. 1).

Because the taxa of the Honggou fauna have not been previously described in the Qaidam basin, it is difficult to directly correlate the Honggou fauna to other fossil assemblages in the region. However, the Olongbuluk mammal fauna discovered from the Huaitoutala section shares taxa with the Tunggur fauna, notably *Lagomeryx* and *Stephanocemas*^15^,^39^, suggesting that all three faunas have similar age ranges. The Tunggur fauna is assigned to the European MN7/8 mammalian zones^34^. In addition, Fang *et al*.^15^ estimated the magnetostratigraphic age of the Olongbuluk fauna as 14.1–12.5 Myr. Given the faunal similarities among these, we conclude that the Honggou fauna is similar in age.

### Honggou magnetostratigraphy

**Figure 4:**
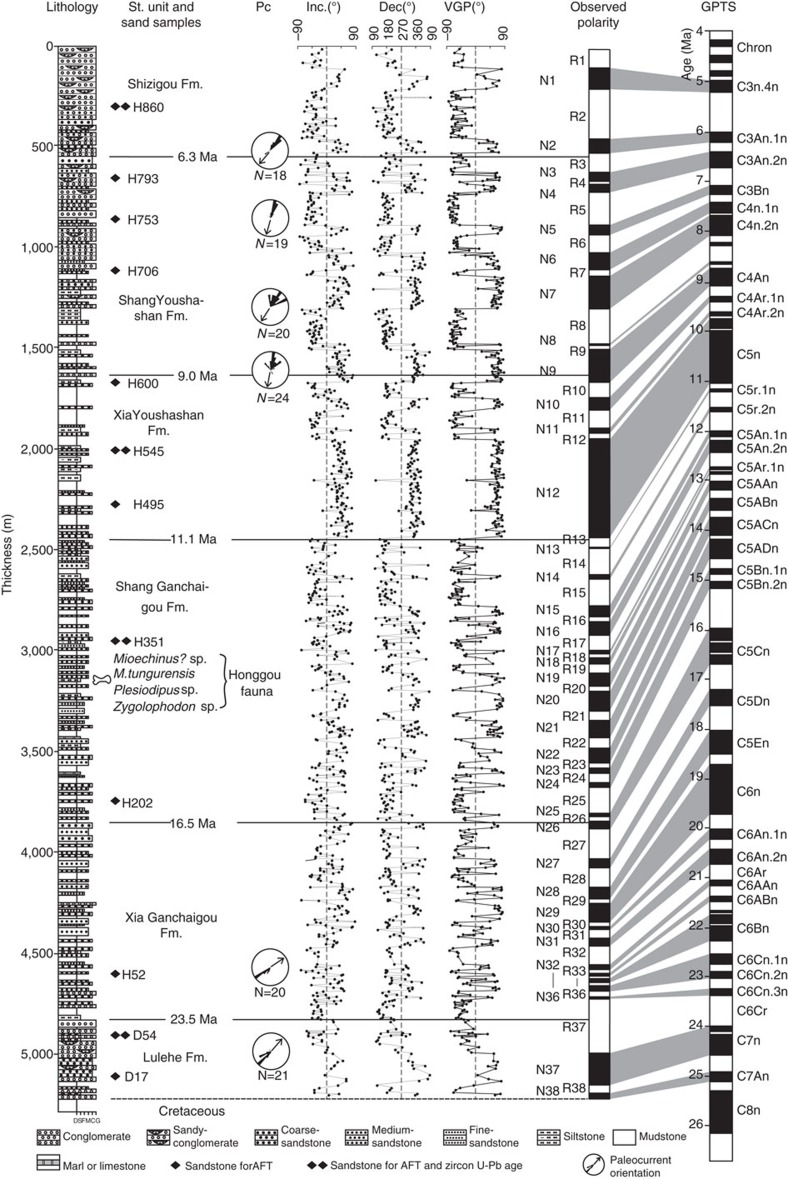
Correlation of the Honggou magnetostratigraphy with the geomagnetic polarity timescale. Sites and main components of fossil mammals found along the section are plotted on the right side of the stratigraphic column for general age constraint. The sandstone samples and paleocurrent indicators also show in the Figure. GPTS, Geomagnetic polarity time scale; Pc, Paleocurrent indicators; VGP, virtual geomagnetic polarity.

After removing secondary viscous remnant magnetizations, 38 pairs of normal and reversed polarity zones (marked as N1–N38 and R1–R38) were identified (Fig. 4). As mentioned above, Honggou fauna was discovered in the magnetozone N19 (at level of 3,158 m) and correlated to the European Land Mammal Zone MN7/8 (ref. 34). Above the Honggou fauna, a strikingly long normal interval N12 can be readily correlated to the characteristic long normal chron C5n of the Geomagntic Polrity Time Scale (GPTS 2012)^40^. The short normal intervals N13-N18 below N12 then likely match C5r.1n-C5Ar.2n. Two closely spaced normal intervals N19 and N20 can be correlated to C5AAn and C5ABn (Fig. 4). Thus, the Honggou fauna suggest an age of about 13.1 Myr for this level within the section, being consistent with 14–12 Myr. Constrained by the Honggou fauna and the long normal interval N12, the long reverse interval R25 and relatively long normal intervals N9, N7 and N1 seem to be correlated to C5Bn.2r, C4An, C4n.2n and C3n.4n, respectively (Fig. 4). The intervals N24–N21 therefore, can be chrons C5Bn.2n-C5ACn; N11–N10 and N6–N2 match also well with chrons C4Ar.1n-C4Ar.2n and C4n.1n-C3An.1n (Fig. 4).

Below the R25, we correlate two short normal intervals of N25 and N26 to chrons C5Cn (Fig. 4). Although the chron of C5Cn in the GPTS^40^ contains three normal polarity zones and one of them is not detected between the N25 and N26 in the Honggou section, alternative correlations of these two normal intervals are practically impossible because the underlying magnetozones of N27-N31 have a strong correlation to the C5Dn to C6An.2n of the GPTS^40^. The interval R32-R36 is characterized by frequent occurrence of short polarity zones, for example, N32, N33, N34, N35 and N36. This pattern of magnetozones appears to correlate with chrons C6ABn to C6Cn.2n of the GPTS^40^, assuming that several short polarity zones are missing. Further below, a correlation between long reverse R37 and C6Cr seems likely. Guided by this, the magnetozones of N37–N38 were correlated to C7n-C7An of the GPTS^40^ (Fig. 4).

Collectively, the correlation of the Honggou magnetostratigraphy to the GPTS implies that the onset of sediment accumulation of the Qaidam basin along the Honggou region occurred at ∼25.5 Myr and that sediment accumulation was continuous to ∼4.8 Myr. Our results imply that the Lulehe Fm. was deposited from 25.5 Myr to 23.5 Myr, the Xia Ganchaigou Fm. was deposited from 23.5 Myr to 16.5 Myr, the Shang Ganchaigou Fm. spans from 16.5 Myr to 11 Myr, the Xia Youshashan Fm. spans from 11–9 Myr, the Shang Youshashan Fm. was deposited from 9–6.3 Myr and the Shizigou Fm. ranges from 6.3 to 4.8 Myr.

Our new chronologic constraints from the Honggou magnetostratigraphy suggest that the onset of Cenozoic sediment accumulation in the northern Qaidam basin is much younger than the previous magnetostratigraphic correlations^20^,^21^,^22^,^23^. The Dahonggou section of Lu and Xiong^20^ is located only ∼10 km east of the Honggou section (Fig. 1) and encompasses the Shang Ganchaigou, Xia Youshashan and Shang Youshashan formations. Lu and Xiong^20^ correlated the Dahonggou magnetic reversal stratigraphy to C13n and C4An of the GPTS based on reports of *Chilotherium*, *Cyprideis*, and *Gomphotherium* in the strata^41^. However, these fossils were first described thirty years ago and uncertainty in their locations hinders the precision of the correlation between the Dahonggou magnetic polarity sequences and GPTS.

**Figure 5:**
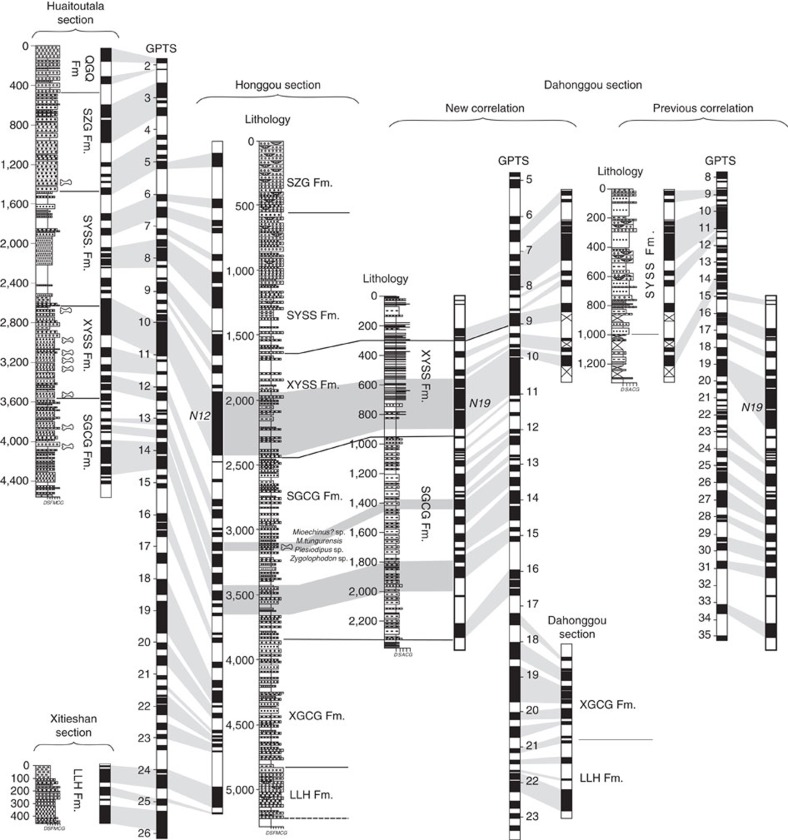
Correlations of stratigraphy and magnetostratigraphy of Cenozoic sedimentary sequences within the Qaidam basin. The revised magnetostratigraphies for Dahonggou section^20^,^21^ and Xitieshan section^22^ based on age control of Honggou fauna and regional stratigraphic correlation. The magnetostratigraphy of all sections indicate a late Oligocene formation of the Qaidam basin.

The proximity of the Honggou and Dahonggou sections allow reasonably confident correlation of the strata containing the Honggou fauna within the Shang Ganchaigou Fm. (level of ∼1,400 m) along the Dahonggou q section (Fig. 1c and Fig. 5). Supporting evidence for this correlation includes: (1) several thin marl beds below the Honggou fauna observed in the Honggou section, that are also found in the Shang Ganchaigou Fm. of the Dahonggou q section (Fig. 5). (2) The Xia Youshashan Fm. includes the finest-grained deposits within the entire Honggou section. This stratigraphic unit correlates well with mudstone-rich Xia Youshashan Fm. in the Dahonggou q section. (3) The longest normal interval in the Honggou section is N12 (∼500 m thick) and is found ∼600 m above the Honggou fauna. There is a prominent ∼400 m thick normal interval present between 550 and 950 m of the Dahonggou q section that appears to represent this same chron. On the basis of the age of the Honggou fauna and the strikingly long normal interval above the fauna, the reversal stratigraphy in the Dahonggou section of Lu and Xiong^20^ appears to correlate with C5Cn to C3An.2n in the GPTS^40^, suggesting that the revised basal age of the strata sampled along the Dahonggou section should be reassigned to ∼16.5 Myr, significantly younger than the previously inferred age ∼34 Myr^20^ (Fig. 5).

East of the Dahonggou section (Fig. 1a), the ∼500 m thick Lulehe Fm. in the Xitieshan magnetostratigraphic section was previously correlated to 51–54 Myr^22^, but the relatively few reversals observed in the section do not allow a definite match to the GPTS. Rather, Xue *et al*.^22^ relied on a broad correlation with pollen^17^ to support this interpretation. Because most pollen in this section reflect flora that span a relatively long time, they do not provide a definitive match for the Xitieshan magnetostratigraphy. The reversal stratigraphy of Xue *et al*.^22^ can also be correlated with C7n to C8n in the GPTS^40^, consistent with an age of 24–26 Myr for the Lulehe Formation (Fig. 5).

Finally, our new interpretation of the age of the Cenozoic strata in the northern Qaidam basin is also consistent with a well-constrained magnetostratigraphic section in the Huaitoutala anticline^15^. Zhuang *et al*.^12^ subdivided the section into six stratigraphic units according to observed lithofacies (Fig. 5). The Huaitoutala magnetostratigraphic section includes the upper five units that may equal to the Qigequan, Shizigou, Shang Youshashan, Xia Youshashan and Shang Ganchaigou formations. Numerous fossil mammals were discovered in the section and provide a robust tie of the reversal stratigraphy from the base to the top of the section^15^. The lowermost Shang Ganchaigou Fm. is estimated to have formed between 15.7–12.5 Myr (Fig. 5); this correlation is consistent with, although a few Myr younger than, the previous interepretaion of Fang *et al*.^15^.

Overall, all of the previous magnetostratigraphic studies along the northern margin of the Qaidam basin are consistent with our new chronology of the Honggou section. Thus, we suggest that the onset of Cenozoic sediment accumulation in the Qaidam basin occurred in Oligocene time, significantly younger than previously thought^6^,^12^,^14^.

### Sediment provenance from detrital thermochronology

Armed with this new chronostratigraphic framework, we use the ages of detrital mineral systems to determine sediment provenance. Previous study of the Honggou section^16^ showed distinct changes up-section in the populations of detrital zircon U-Pb ages; these appear to reflect changes through time from a single source region along the southern margin of the Qaidam basin (the East Kunlun Shan) early in the depositional history (Lulehe Fm.) to the addition of material from the Qilian Shan higher in the section (Xia Ganchaigou Fm.). Here we add analyses of detrital apatite fission-track ages. Because these ages reflect cooling of source terranes through annealing temperature of ∼120 °C, they provide a complementary means of evaluating source region, but also place bounds on the rate of erosion and transport from the range to the basin.

**Figure 6:**
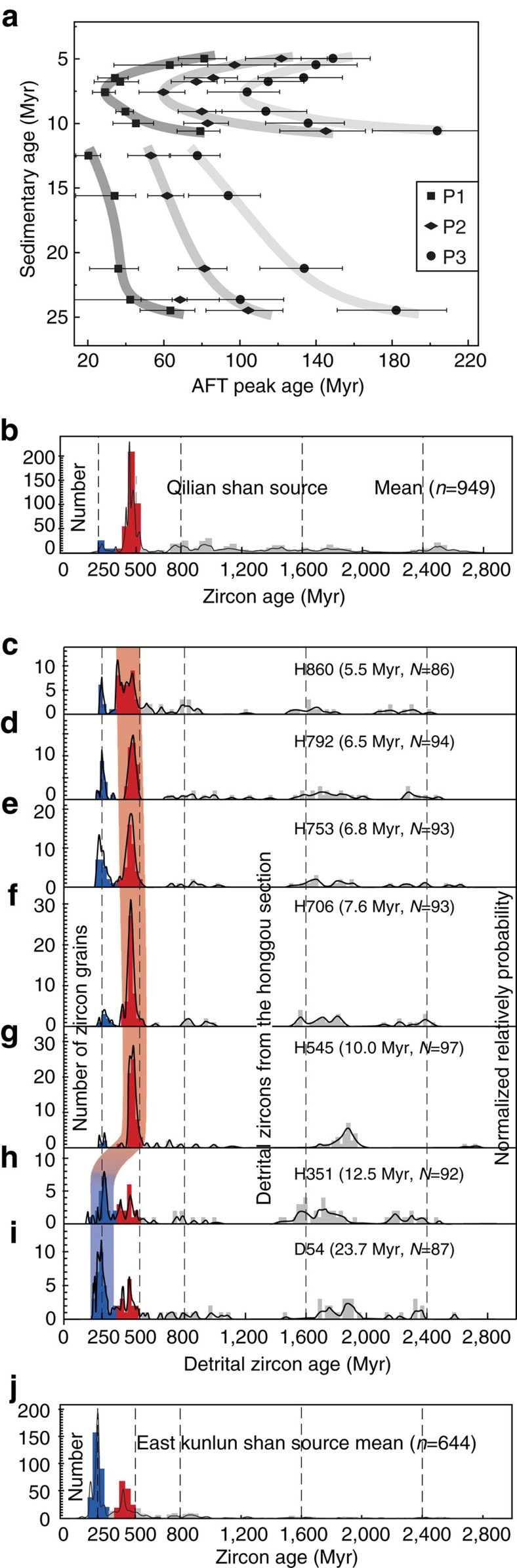
Provenance data from the Honggou section and potential source regions. (**a**) Apatite fission track (AFT) population peak ages plot for samples from the Honggou section. Error bars are ±2σ for apatite FT ages. P1, P2 and P3 represent the youngest, second youngest and third population peak ages, respectively. The dark to light lines show the youngest, second youngest and third population peak ages trend with sedimentary ages of the samples. (**b**–**j**) Zircon U-Pb age cumulative probability distributions from the Qilian Shan (**b**), East Kunkun Shan (**j**) and the Honggou section (**c**–**i**). For the zircon U-Pb age plots, the blue rectangles are 180–350 Myr ago zircon grain histograms, whereas the red rectangles are 350–500 Myr ago zircon grain histograms. The colour change from blue to red in vertical shade areas represents source variation. The sedimentary ages of the samples are based on the Honggou magnetostratigraphy.

The binomial peak fitting method^42^ (see methods section) was used to decompose the thirteen measured detrital AFT ages into statistically significant age populations (Supplementary Table 2). We rule out partial resetting for these AFT ages, because all of the samples have relatively long fission-tracks ranging from 11.8 to 12.3 μm (Supplementary Fig. 2) and because the youngest population peak ages are considerably older than their respective depositional ages (Supplementary Table 2, Supplementary Fig. 3). Therefore, we conclude that the apatite grains in the samples preserve cooling episodes in the source rocks. In our section, the youngest (P1), second youngest (P2) and third (P3) population peak ages have a distinct trend: except for D54, the samples with depositional ages older than 12.5 Myr show gradually decreasing peak ages upsection; P1, P2 and P3 decrease from 63.4, 104.4, 182.1 Myr at the base of the section to 20.3, 53.2 and 77.6 Myr near the ∼12 Myr boundary. Peak ages sharply increase to 79.1, 145.2 and 203.7 Myr at 10.6 Myr, and then show progressively decreasing peak ages followed by another increase. The minimum peak ages occur near ∼7.5 Myr (Fig. 6a).

The sharp increase for the population peak ages at ∼12 Myr require an abrupt change in sediment provenance, because progressive unroofing of a single source region will yield systematically younger ages upsection as exhumation drives cooling of rocks. The mirror-image distribution peak ages in the upper part of the section probably represent deposits in the section containing first-cycle detritus after 7.5 Myr ago.

These interpretations are supported by detrital zircon provenance analysis. In addition to the previous work by Bush *et al*.^16^, we collected seven samples for U-Pb analysis of zircons from samples co-located with the apatite-fission track results (Fig. 4). The detrital-zircon U-Pb age analytical results are listed in the Supplementary Data 1, and these ages are compared with zircon ages in the East Kunlun Shan and Qilian Shan, which serve as the two most straightforward source regions of the basin (Fig. 1a).

In our Honggou section, two pre-12.5 Myr samples (D54, H351) exhibit similar detrital-zircon age spectra. Zircon ages in the both sample are distributed between 184 and 2,581 Myr, with a major 250 Myr peak and three minor peaks at 440, 1,750 and 2,350 Myr (Fig. 6h-i). Different with the age distributions of the pre-12.5 Myr samples, zircon grains in the post-10.5 Myr samples are dominated by age probability peaks at ∼440 Myr, which account for ∼55% of the total dated grains in the samples H545, H706 and ∼40% in the samples H753, H792 and H860 (Fig. 6c-g). Grains younger than 350 Myr and older than 500 Myr comprise <10% and <35% in the post-10.6 Myr samples.

Along the northern Qaidam basin margin, granite bodies in the Qilian Shan are dominantly early Paleozoic in age^43^,^44^, with few of the Permo-Triassic granitic plutons^41^ (Fig. 1a). Correspondingly, the detrital zircons eroded from the Qilian Shan were characterized by a prominent age population between 410–510 Myr (Fig. 6b). However, Permo-Triassic granitic bodies widely crop out along the East Kunlun Shan, with a prominent zircon age population between 200–300 Myr ago^43^,^45^ (Fig. 1a, Fig. 6j). The Proterozoic zircons seem to be preserved in the Proterozoic rocks that are exposed both in the East Kunlun Shan and the Qilian Shan. Comparison of zircon age distributions between Qaidam basin fill, East Kunlun Shan and Qilian Shan shows similarity between the pre-12.5 Myr ago samples and source regions in the East Kunlun Shan, whereas the post-10.6 Myr ago samples are similar to regions in the Qilian Shan. These results are consistent with the interpretations of Bush *et al*.^16^ and suggest that the pre-12.5 Myr deposits were likely derived from regions in East Kunlun Shan, and that the post-10.6 Myr ago sediments were dominated by influx of material from the Qilian Shan region. Regional analyses show similar patterns in the Huaitoutala section^16^, suggesting that this shift reflects regional patterns of mountain building along the margins of the Qaidam Basin.

Our detrital AFT and the detrital-zircon age distribution results are also supported by paleocurrent data. The north- to northeast-directed paleocurrent indicators in the lower part of the section are generally consistent with the source of the East Kunlun Shan and the appearance of south-directed paleocurrent orientations in the upper part of the magnetostratigraphic section provides an independent evidence of the Qilian Shan derived material entering the Qaidam basin since the late Miocene time (Fig. 4).

## Discussion

**Figure 7:**
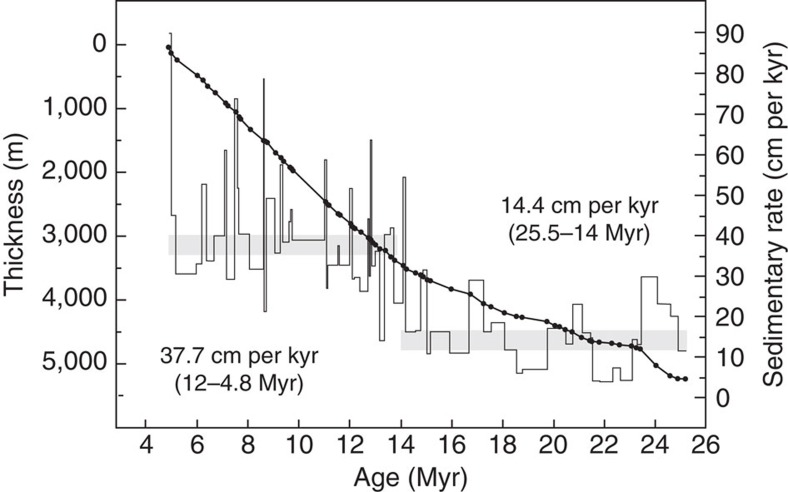
Age versus thickness plots of the Honggou section. Sediment accumulation rates are also plotted as well. The horizontal shaded zones represent the average accumulation rates. The sedimentary age data are based on the Honggou magnetostratigraphy.

Our new chronology from the Honggou reversal stratigraphy significantly revises the timing of sediment accumulation in Qaidam basin during the Cenozoic. The onset of deposition (Lulehe Fm.) occurred around ∼25.5 Myr and persisted until ∼4.8 Myr. Accumulation rates were relatively steady through the interval between ∼25.5–14 Myr at ∼14.4 cm per kyr (Fig. 7). Sometime between 14–12 Myr, accumulation rates increased to ∼37.7 cm per kyr (Fig. 7). The increase in accumulation rates appears to be coincident with shifts in sediment provenance around ∼12 Myr and probably reflects the influence of sediment derived from the newly emergent Qilian Shan. The timing of these events challenge the consensus interpretation that initial uplift and development of high topography adjacent to the Qaidam basin initiated during, or prior to, Eocene time.

First, the new chronology of the Honggou section reveals that the onset of Cenozoic sediment accumulation in the Qaidam basin occurred at ∼25.5 Myr ago, which is significantly younger than the previous interpretations which held that deposition of the coarse detritus in the Lulehe Fm. occurred between 65–50 Myr ago^6^. As we argue above, all of the existing chronologic bounds on the age of the Lulehe Fm. along the northern margin of the Qaidam Basin are consistent with this revised age interpretation. Basin fill in the north basin margins appears to have been derived from the East Kunlun Shan at this time^16^, suggesting that the East Kunlun Shan emerged as a high relief margin along the southern boundary of the Qaidam basin at the late Oligocene. We note that this timing is consistent with the existing bedrock thermochronologic data, which indicate exhumation and rock uplift in the East Kunlun Shan starting at ∼30 Myr^46^,^47^,^48^. Thus, our revised age for the Luluhe Fm. reconciles previously incompatible proxy data for the timing of source area exhumation and basin deposition. Taken together, those observations present a compelling argument that Oligocene uplift of the East Kunlun Shan along the East Kunlun reverse fault was the primary driver of flexural subsidence in the Qaidam basin during the mid-Cenozoic. It seems likely that crustal shortening along the southern margin of the Qaidam basin marked the northern paleo-boundary of the Tibetan Plateau during the Oligocene.

Second, our results indicate that no significant Paleogene tectonic deformation occurred in the region north of the East Kunlun Shan. The Honggou magnetostratigraphic section is adjacent the Qilian Shan, which separates the Qaidam basin from the Hexi Corridor basin in the north. The absence of Qilian Shan derived materials in the pre-middle Miocene deposits, as revealed by the detrital AFT ages and detrital zircon ages (Fig. 6), requires the Qilian Shan to have had relatively low topographic relief during Oligocene to middle Miocene. We infer that these regions north of the Qaidam basin were tectonically quiescent at this time. In addition, the remnants of mid-Tertiary sedimentary rocks (notably the Shang Ganchaigou and Xia Youshashan formations) are widespread across the South Qilian Shan region, and the lacustrine- fluvial facies associations suggest deposition in a broad basin with few local depocenters^49^. Assuming that these sediments were once continuous with similar deposits in the Qaidam basin, it seems likely that present day topography that characterizes the South Qilian Shan must have developed subsequent to the mid-late Miocene (∼12 Myr). Our results therefore directly contradict previous studies^6^,^12^ that infer significant crustal shortening across the Qilian Shan region during Paleocene to Eocene.

Third, our results support the conclusion that significant tectonic deformation and uplift of the Qilian Shan began at 14–12 Myr. The key results from the Honggou magnetostratigraphy and provenance analysis are the significant increase in accumulation rates at 14–12 Myr and abrupt shift to a sediment source north of the Qaidam basin at this time. These changes are interpreted to reflect the onset of rapid uplift of the Qilian Shan during the late Miocene. Notably, a similar shift in sediment provenance is observed along the northern margin of the Qilian Shan, in the Hexi Corridor at ca.12 Myr^50^ and thermochronology from the northern portion of the range suggest that onset of rapid exhumation occurred at ca. 10 Myr ago^51^. Although climate change could contribute to increased erosion and sedimentation rates at this time^52^, simultaneous shifts in the provenance of sediment accumulated along both the southern and northern margins of the Qilian Shan argue strongly for the emergence of high topography at this time. Thus, in our interpretation, most of the crustal shortening across the Qilian Shan^53^ accumulated since the late Miocene.

Our refined ages for sediment deposition as a proxy for mountain building in northern Tibet carry important implications for the geodynamics of plateau growth. The onset of deformation along the East Kunlun Shan at ∼25.5 Myr, accompanied by relative tectonic quiescence of the Qilian Shan, suggests that shortening related to the Indo-Asian collision during Eocene time was restricted to the Hoh Xil region, south of the East Kunlun Shan^54^. Significant deformation did not extend far beyond the East Kunlun Shan thrust system. Synchronous emergence and rapid exhumation of both the northern and southern margins of the Qilian Shan at ∼12 Myr^15^,^49^,^50^,^51^ suggest that the modern extent of Cenozoic deformation and high topography along the northeastern Tibetan Plateau was established at this time. Moreover, evidence for the onset of range growth during the late Miocene is widespread across regions of northeastern Tibet^55^,^56^,^57^. Regional synchronicity of mountain building across thousands of kilometers of the continental interior suggests a system-wide change in the dynamics of mountain building during the late Miocene. Thus, our results reinvigorate the debate over changes in the lithospheric buoyancy in northern Tibet^1^,^58^, could have driven widespread deformation and crustal thickening outboard of the plateau. We suggest that high topography, crustal shortening, and the continued active deformation of the Qilian Shan and northeastern Tibet may be a consequence of this process.

## Methods

### Magnetostratigraphy

A gasoline powered drill was used to collect the magnetostratigraphic samples at intervals of 4–6 m throughout the section and at least three cores per were drilled. In total, 2,970 cores were collected from 990 stratigraphic horizons. All the drilled cores were oriented by a magnetic compass corrected ∼1.8° to account for the local magnetic declination anomaly.

All 2,970 core samples (990 × 3 pilot samples) were shorten to standard specimens of 2 cm in length in the laboratory. At last one specimen per site was subjected to stepwise thermal demagnetization in a TD-48 thermal demagnetizer with an internal residual field less than 10 nT. The maximum of 20 steps for thermal demagnetization were applied for the samples with the following stepwise heating routine: 20, 150, 200, 250, 300, 350, 400, 450, 500, 525, 550, 585, 610, 620, 630C, 640, 650, 660, 680 and 690 °C. The magnetic remanence of each sample was measured with a 2G Enterprises Model 760, three-axis, cryogenic magnetometer shielded in field-free space (<300 nT), at the Paleomagnetism Laboratory of the Institute of Geology and Geophysics, Chinese Academy of Sciences.

The intensity of the natural remanent magnetization (NRM) of the Honggou section samples was typically on the order of 10^−2^ A/m, with a range of 10^−1^–10^−3^ A/m. Progressive thermal demagnetization successfully resolved multiple components of magnetization (Supplementary Fig. 4). Most samples possess two magnetic components: a low-temperature component and a high-temperature component (Supplementary Fig. 4). The low temperature component is typically removed by 200–250 °C, but sometimes not until 450 °C. This component is interpreted to be as a viscous overprint by modern magnetic field. The high-temperature component decays towards the origin, typically exhibits stable behaviour between 450–680 °C, and is interpreted to reflect the characteristic remanent magnetization (ChRM). Complete unblocking of the high-component by 680 °C indicates that hematite is the carrier of the magnetization in the section, but the presence of magnetite is suggested as well by an accelerated decay of the magnetization at 585 °C (Supplementary Fig. 4). In the section, there is no significant difference in remanent direction, when it is defined by the 450–585 °C or 600–680 °C parts of the unblocking temperature spectra. This suggests that both magnetic carriers recorded the same paleomagnetic field. We used least-squares, principal-component analysis^59^ to isolate the ChRM directions for each specimen. Some samples were rejected when they (1) could not be determined the ChRM; (2) can be revealed the ChRM directions, but the maximum angular deviation >15°. Finally, 770 (78%) samples from the Honggou section gave reliable ChRM directions (Supplementary Data 2).

A reversal test^60^ however, is positive with an angular difference that is less than the critical angle and yields a C classification reversal test (Supplementary Fig. 5). Further analysis using Tauxe and Watson^61^ fold test presents an optimal concentration at 102% unfolding with the 95% uncertainties ranging from 90 to 114% untilting defining a positive fold test (Supplementary Fig.6).

### Apatite fission track dating

Thirteen medium- to coarse-grained sandstone samples were collected from the Honggou section to analyse their provenance. More than 5 kg of sandstones for each of the samples were collected from a single outcrop. The apatite and zircon crystals in the samples were extracted using the following methods: (1) the sandstone samples were first crushed to pass a 60 mesh (250 μm) sieve in the laboratory. (2) Manual washing with water and then alcohol was performed to get the denser component of various grain sizes. (3) Electrometer was used to remove magnetic minerals. (4) Different density heavy liquids were employed to separate the apatite and zircon crystals, respectively. (5) The apatite and zircon grains are checked and some non-apatite or non-zircon grains are picked out by hand under a binocular.

The external detector method^62^ and a zeta calibration factor^63^ determined from the Durango apatite and Fish Canyon apatite were employed to obtain the Apatite fission track ages. Apatite mounts were irradiated at the 492 reactor, Institute of Atomic Energy of China and apatite fission track analysis was carried in the Institute of Geology, China Earthquake Administration. Spontaneous fission tracks in apatite were etched in 5.5% HNO_3_ at 20 °C for 20 min. Induced fission tracks in the low-U muscovite external detectors, which covered apatite grain mounts and glass dosimeters during the irradiation were later etched in 40% HF at 20 °C for 20 min. Fission tracks and track length measurements were measured on an OLYMPUS microscope using a magnification of 1,000 under oil immersion objectives for apatite. Generally, the detrital apatite fission track age represents mixed multiple component ages. We therefore employ the binomial peak-fit method^42^ to get the component ages. The binomial peak-fit method is a maximum-likelihood procedure to find a best solution assuming binomially-distributed components^42^.

### Zircon U-Pb dating

Detrital zircon grains were dated by laser ablation inductively coupled plasma mass spectrometry in the Institute of Geology and Geophysics, Chinese Academy of Sciences, following the standard procedure^64^. The Harvard zircon 91500 was used as standard to correct for mass bias affecting ^207^Pb/^206^Pb, ^207^Pb/^235^U, ^206^Pb/^238^U (^235^U =^238^U/137.88) ratios. Standard NIST 610 silicate glass was used for concentration information and the Th/U ratio determination. Analyses with ≥20% discordance or ≥5% reverse discordance were excluded. We adopt the ^206^Pb/^238^U ages for grains younger than 1,000 Myr and ^207^Pb/^206^Pb ages for grains older than 1,000 Myr. The detrital zircon age populations for individual sample are plotted on relative age-probability diagrams derived from the probability density function.

### Paleocurrent determination

Paleocurrent orientations were primarily determined from clast imbrication and cross stratification along the Honggou section. All the orientation data were measured by a magnetic compass corrected ∼1.8° to account for the local magnetic declination anomaly. For each site, at last 18 imbricated clasts or cross stratifications are measured. The measured data just have been correlated for tilted bedding because the paleomagnetic data^13^,^65^, geological mapping^41^ and Global positioning system measurements^66^ suggest that no postdepositional rotation took place in the Qaidam basin.

### Data availability

The authors declare that all data supporting the findings of this study are available within the paper and its Supplementary Information files.

## Additional information

**How to cite this article:** Wang, W. *et al*. Expansion of the Tibetan Plateau during the Neogene. *Nat. Commun.* **8,** 15887 doi: 10.1038/ncomms15887 (2017).

**Publisher’s note:** Springer Nature remains neutral with regard to jurisdictional claims in published maps and institutional affiliations.

## Supplementary Material

Supplementary Information

Supplementary Data 1

Supplementary Data 2
