# Supplementary material for: Expansion of the Tibetan Plateau during the Neogene
Source: Nat Commun. 2017 Jun 21;8:15887. doi: 10.1038/ncomms15887 (PMC5482058; doi:10.1038/ncomms15887)
Supplement: Supplementary Information [file ncomms15887-s1.pdf]

File name: Supplementary Information
Description: Supplementary Figures, Supplementary Tables and Supplementary References

File name: Supplementary Data 1
Description: Detrital zircon U-Pb ages of the sandstone samples from the Honggou section.

File name: Supplementary Data 2
Description: Accepted characteristic remanent magnetization (ChRM) directions of the paleomagnetic samples from the Honggou section.

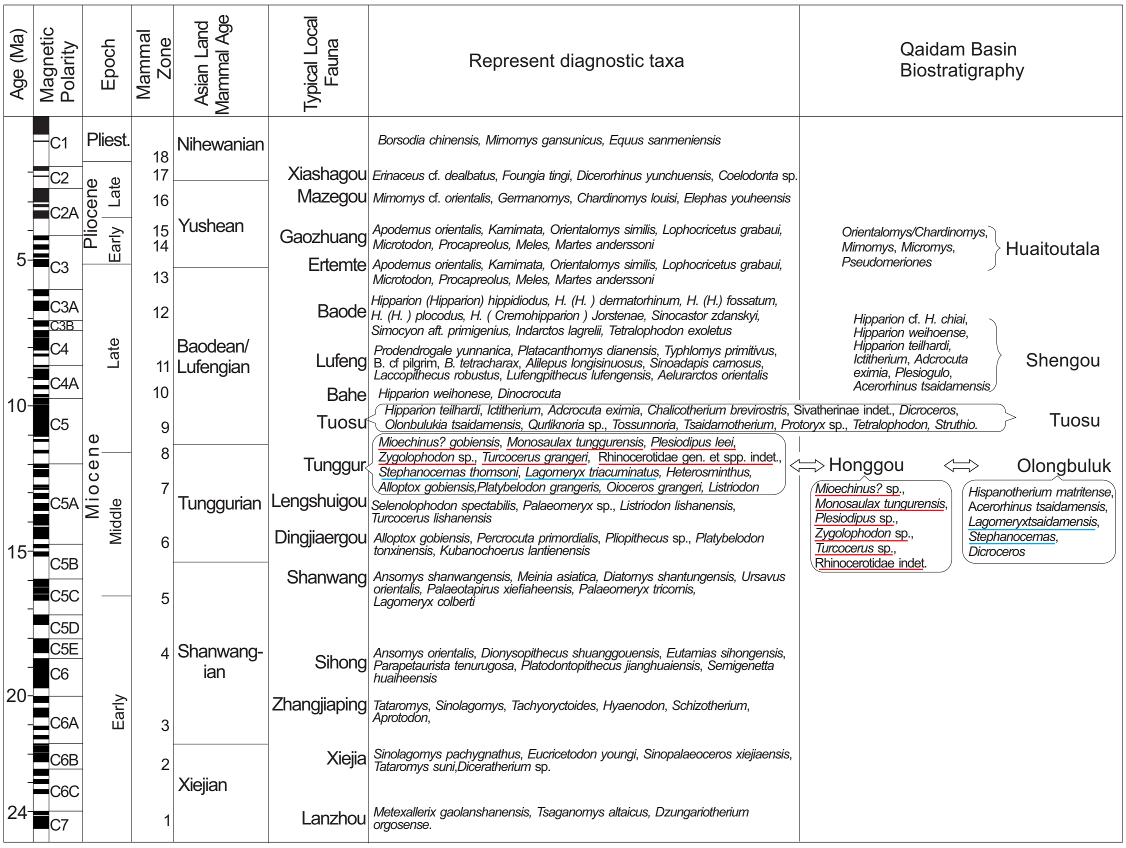

Supplementary Figure 1 Correlations of the Honggou fauna to the Chinese terrestrial Neogene faunas divided by Qiu and Qiu[1] and to the Qaidam basin mammal faunas[2]. All six taxa of the Honggou fauna share similar characteristics with corresponding fossils (marked out by red lines) from the middle Miocene Tunggur fauna in Inner Mongolia, but have little in common with the ealy-middle Miocene Lengshuigou fauna and late Miocene Tuosu faunas. The Olongbuluk fauna[2] discovered in the Qaidam basin shares taxa (labelled by blue lines) with the Tunggur fauna.

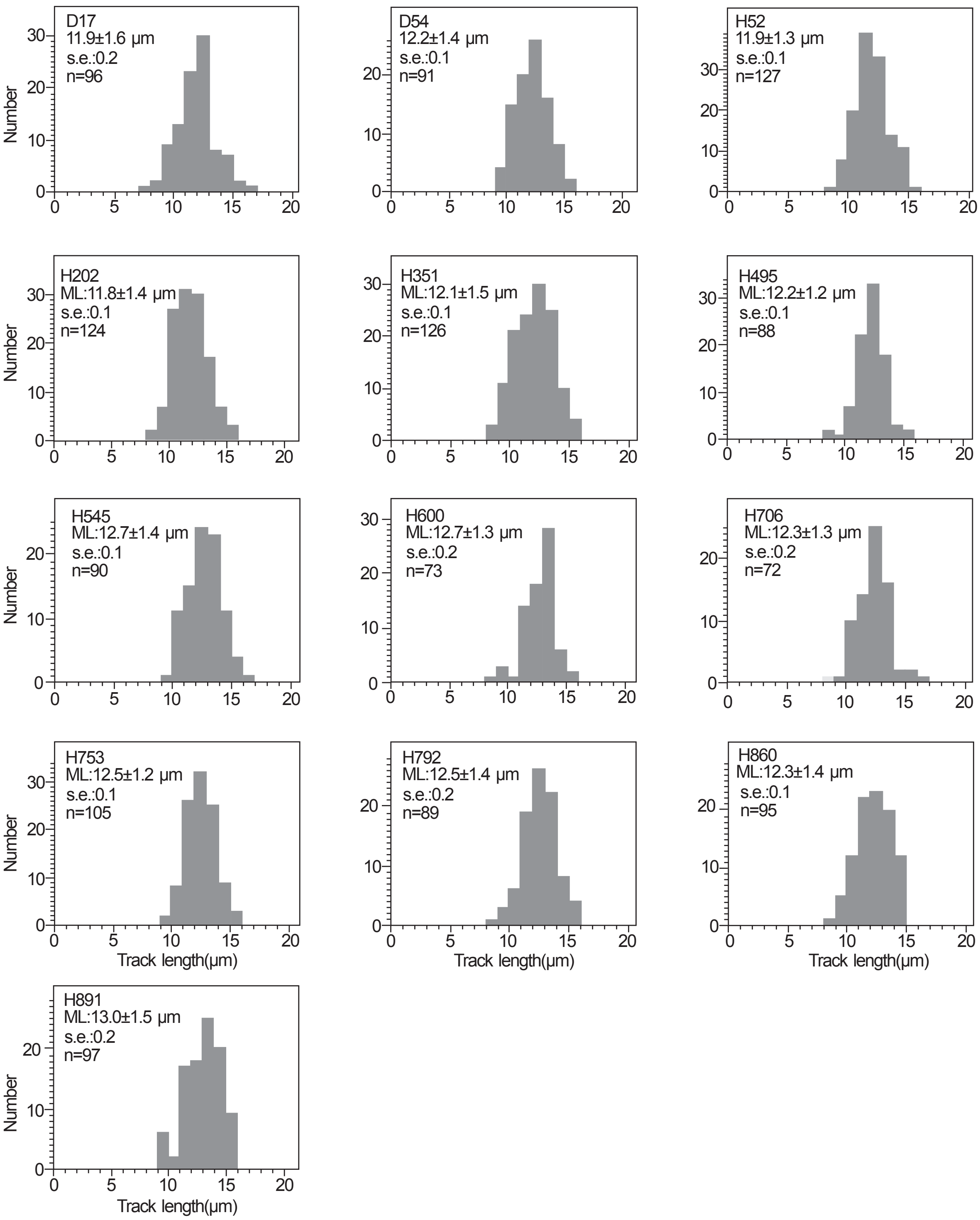

Supplementary Figure 2 Length distributions of fission tracks for the sandstone samples collected from the Honggou section. ML: mean confined track length; s.e.: standard 1σ error of track length; n: number of measured fission tracks.

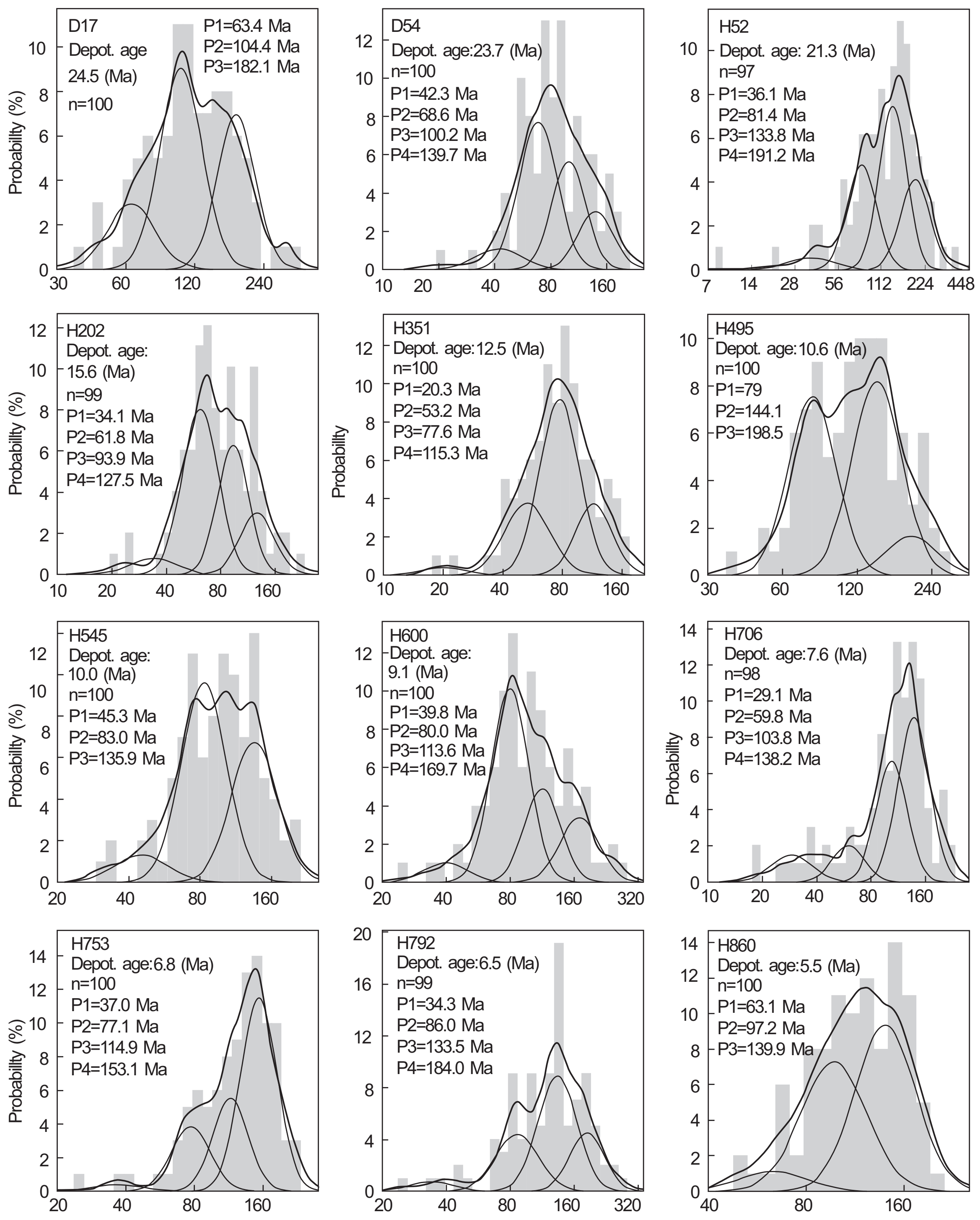

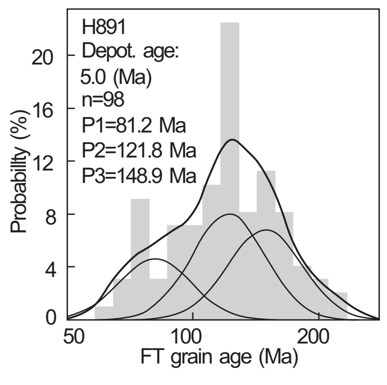

Supplementary Figure 3 Probability density plots of the detrital apatite fission-track samples from the Honggou section. For each sample, the depositional age, number of counted grains and fitted age-peaks are given. Binomial peak fitting was done with Binom Fit program[3].

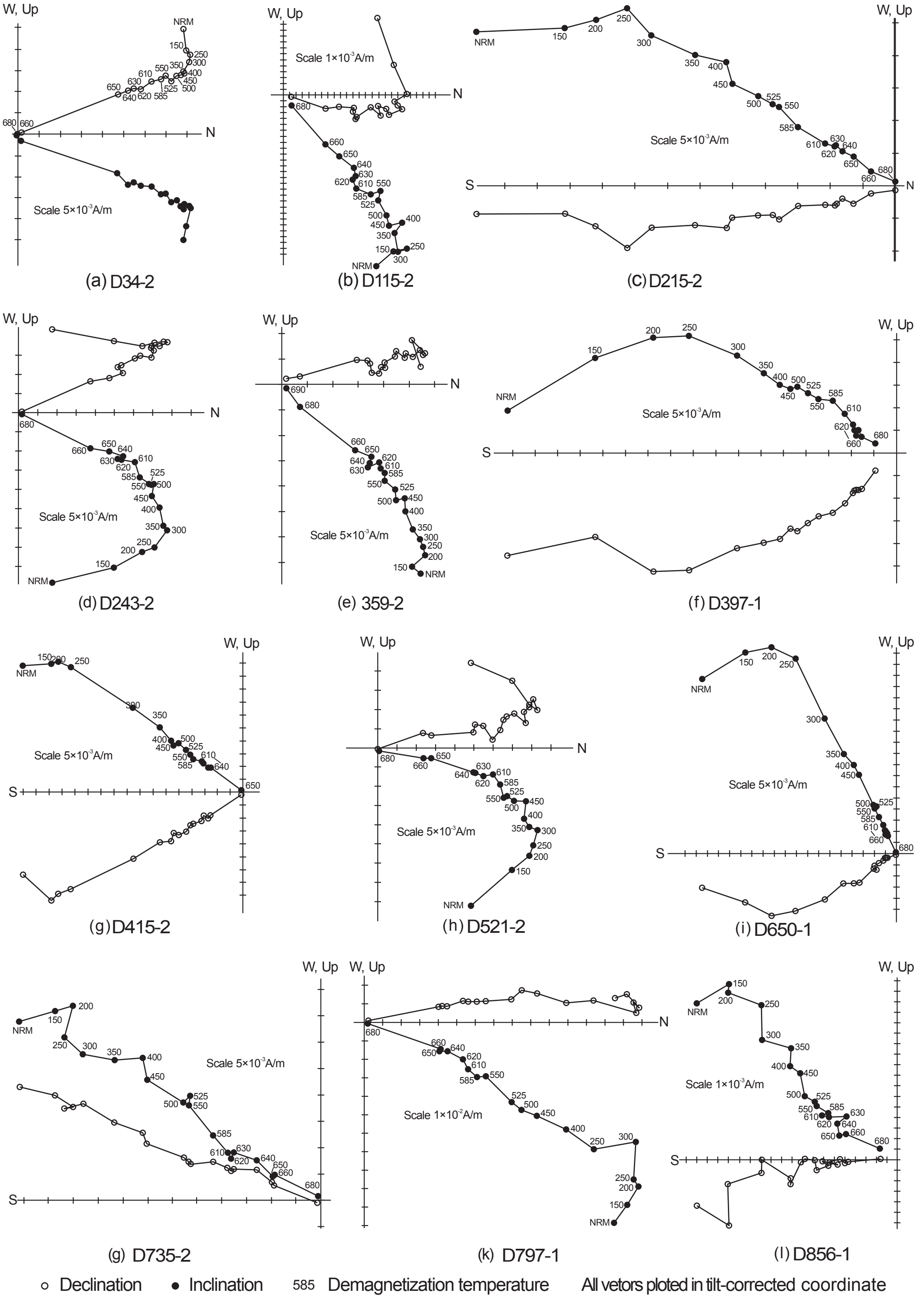

Supplementary Figure 4 Orthogonal (Zijderveld[4]) vector plots of representative thermal demagnetization behaviors of specimens from the Honggou section (a-l). Hollow (Solid) circles plot declination (inclination) within the orthogonal demagnetization diagrams. NRM is the natural remanent magnetization before demagnetization and the numbers mark the steps temperature of demagnetization.

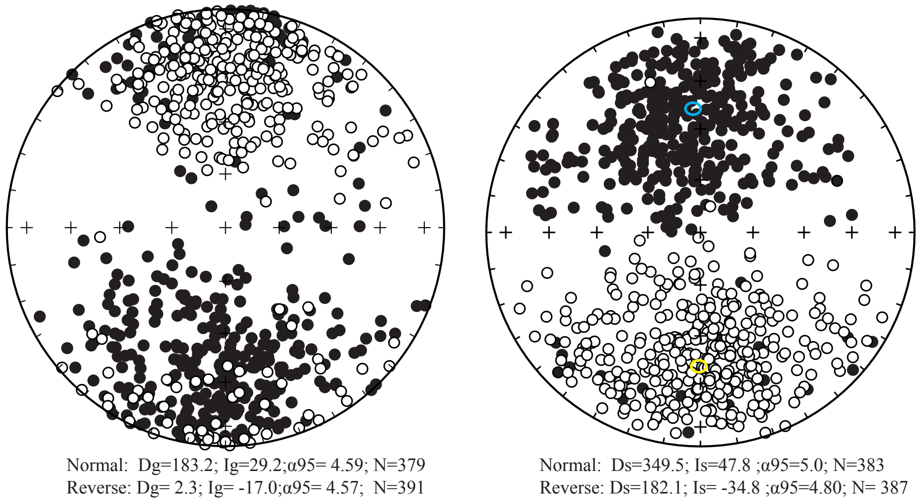

Supplementary Figure 5 Equal-area plots of accepted ChRMs (770 sites) from the Honggou section in geographic and tilt-corrected coordinates, respectively. Hollow (Solid) circles plot in the lower (upper) hemisphere, Ovals indicate α95 error around the Fisher mean with mean data.

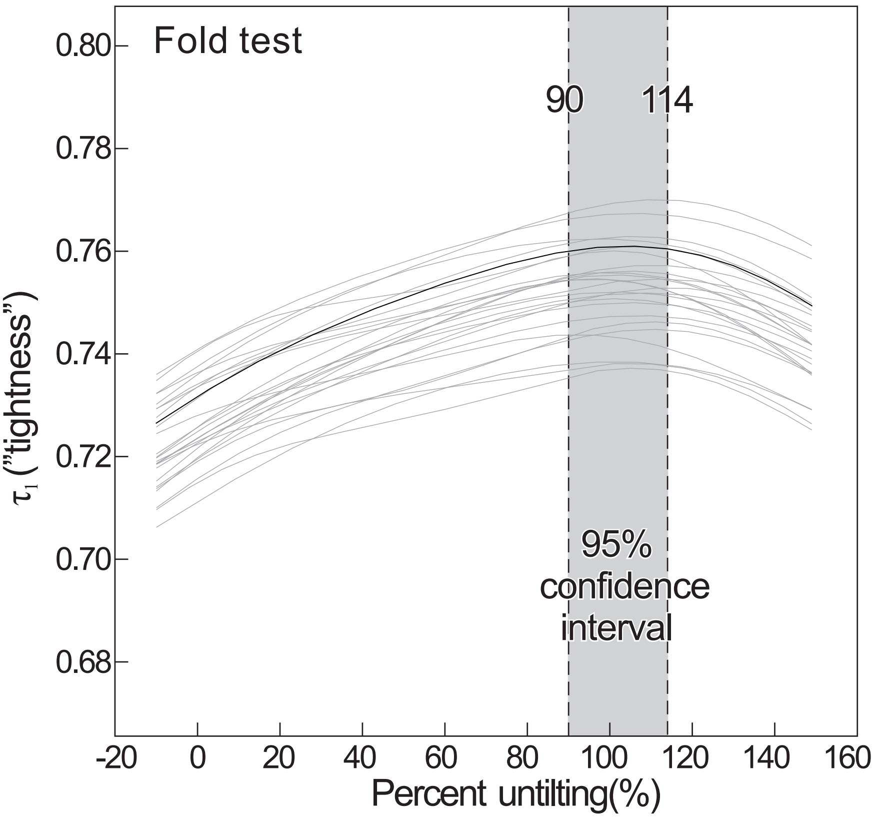

Supplementary Figure 6 Positive fold test for the Honggou characteristic remanent magnetization (ChRM) directions. τ_1 maximum reflects the tightest grouping of the ChRM directions during progressive untilting of the strata and is defined by the eigenvalues of the orientation matrix[5]. The 95% confidence interval for τ_1 overlapping with 100% untilting of ChRM vectors corrected for strata orientation.

Supplementary Table 1 Lithofacies and interpretations used in this study

Code	Description	Interpretation
Fl	Very fine-grained sandstone to siltstone, mudstone with fine lamination	Suspension-settling in ponds and lakes
Fm	Massive, very fine-grained sandstone to siltstone, mudstone	Suspension settling in lake and overbank deposits
Sm	Massive fine- to coarse-grained sandstone	Sandy mud flows and suspension settling in lake and overbank deposits
St	Fine- to very coarse-grained sandstone with trough cross-stratification	Migration of large 3D ripples (dunes) under moderately powerful unidirectional flows in large channels
Sh	Fine- to coarse-grained sandstone with planeparallel lamination	Upper plane bed conditions under unidirectional flows, either strong or very shallow
Sr	Fine- to medium-grained sandstone with small, 2D and 3D current ripples	Migration of small 2D and 3D ripples under weak, unidirectional flows in shallow channels
Cmm	Massive, matrix-supported pebble to cobble conglomerate, poorly sorted, disorganized, unstratified	Deposition by cohesive mud-matrix debris flows
Cci	Pebble to cobble conglomerate, clast-supported, horizontally stratified, imbricated, poorly sorted	Deposition from shallow traction currents in longitudinal bars and gravel sheets

Supplementary Table 2 Detrital apatite fission track results for samples in this study. For sample locations and sedimentary age information, please refer to Figure 4

Sample	Sediment Age/Ma	N	Age range (Ma)	P1			P2			P3			P4		
				Peak age	F	W	Peak age	F	W	Peak age	F	W	Peak age	F	W
H891	5.0	98	64.4-219.3	81.2±13.8/11.8	*23.3*	6.6	121.8±19/24.7	*41.2*	30.1	148.9±16.9/19.6	*35.4*	31.2			
H860	5.5	100	48.6-193.7	63.1±29.7/20.2	*6.7*	8.2	97.2±27.8/21.6	*42.6*	12.5	139.9±23.7/20.3	*50.7*	15.5			
H792	6.5	99	24.4-280.2	34.3±8.9/7.1	*4.6*	2.2	86±15.3/13	*26.1*	6.7	133.5±24/20.4	*47.6*	10.2	184±34.2/28.9	*21.6*	9.9
H753	6.8	100	26.2-227.6	37.0±13.7/10.0	*2.4*	1.6	77.1±13.4/11.4	*18.9*	5.5	114.9±22.8/19	*25.0*	10.0	153.1±22.2/19.4	*53.7*	10.2
H706	7.6	98	19.2-220.3	29.1±6.8/5.5	*9.8*	3.5	59.8±13.1/10.8	*10.4*	4.0	103.8±20.7/17.3	*33.1*	13.2	138.2±23.2/19.9	*46.6*	1.6
H600	9.1	100	25.2-265.0	39.8±5.1/4.5	*6.1*	2.8	80±12.5/10.8	*51.5*	9.6	113.6±26.5/21.5	*24.1*	8.9	169.7±31.1/26.4	*18.2*	5.9
H545	10.0	100	31.8-203.3	45.3±12.2/9.6	*8.3*	3.6	83±12.8/11.1	*53.3*	8.2	135.9±22.5/19.3	*38.4*	1.6			
H495	10.6	100	39.0-261.7	79±12.1/10.5	*42.3*	6.8	144.1±24.5/21	*47.7*	10.0	198.5±34.3/29.3	*10.0*	8.8			
H351	12.5	100	18.0-169.6	20.3±7.4/5.4	*2.3*	1.6	53.2±12.1/9.9	*24.6*	10.1	77.6±14.5/12.2	*53.2*	11.4	115.3±24.9/20.5	*19.9*	9.3
H202	15.6	99	20.1-219.8	34.1±20.8/12.9	*5.7*	4.6	61.8±10.3/8.8	*46.5*	7.9	93.9±20.7/17.0	*31.3*	11.9	127.5±40.5/30.8	*16.5*	12.1
H52	21.3	97	8.2-363.2	36.1±14.9/10.6	*5.2*	2.8	81.4±13.7/11.8	*28.5*	6.4	133.8±23.4/20.0	*41.7*	11.0	191.2±41.7/34.3	*24.6*	10.5
D54	23.7	100	19.8-197.6	42.3±45.3/21.9	*7.8*	13.6	68.6±23.2/17.8	*45.7*	11.2	100.2±29.5/22.8	*29.7*	13.9	139.7±32.7/26.6	*16.8*	9.0
D17	24.5	100	37.9-313.3	63.4±16/12.8	*17.4*	7.2	104.4±22.2/18.3	*48.9*	7.5	182.1±31/26.5	*33.8*	8.3			

Notes Binomial peak-fit ages (P1-P4) were determined with BINOMFIT described by Brandon[3] and are given with 95% confidence interval (note that these intervals are asymmetric), F is the fraction of the specific peak in percent; N, total of grains counted; W, relative standard deviation for a peak. Samples were dated with the external detector method with a zeta (CN5) of 253± 10 yr/cm^2

Supplementary References

1 Qiu Z.X., & Qiu. Z.D. Chronological sequence and subdivision of Chinese Neogene. *Palaeogeography, Palaeoclimatology, Palaeoecology* **116**, 41-70 (1995).

2 Wang, X. et al. Vertebrate paleontology, biostratigraphy, geochronology, and paleoenvironment of Qaidam Basin in northern Tibetan Plateau. *Palaeogeography, Palaeoclimatology, Palaeoecology* **254**, 363-385, (2007).

3 Brandon, M. T. Decomposition of mixed grain age distributions using BINOMFIT. *On Track* **24**, 13-18 (2002).

4 Zijderveld, J. AC demagnetization of rocks: analysis of results. *Methods in paleomagnetism* **3**, 254 (1967).

5 Tauxe, L. & Watson, G. The fold test: an eigen analysis approach. *Earth and Planetary Science Letters* **122**, 331-341 (1994).
